# Using machine learning approaches to predict timely clinic attendance and the uptake of HIV/STI testing post clinic reminder messages

**DOI:** 10.1038/s41598-022-12033-7

**Published:** 2022-05-24

**Authors:** Xianglong Xu, Christopher K. Fairley, Eric P. F. Chow, David Lee, Ei T. Aung, Lei Zhang, Jason J. Ong

**Affiliations:** 1 Central Clinical School, Monash University, Melbourne, Australia; 2 Melbourne Sexual Health Centre, The Alfred, Melbourne, 3053 Australia; 3 Centre for Epidemiology and Biostatistics, Melbourne School of Population and Global Health, The University of Melbourne, Melbourne, VIC Australia; 4 China Australia Joint Research Center for Infectious Diseases, School of Public Health, Xi′an Jiaotong University Health Science Centre, Xi′an, Shaanxi China; 5 Department of Epidemiology and Biostatistics, College of Public Health, Zhengzhou University, Zhengzhou, Henan China; 6 Faculty of Infectious and Tropical Diseases, London School of Hygiene and Tropical Medicine, London, UK

**Keywords:** Infectious diseases, Health services, Public health

## Abstract

Timely and regular testing for HIV and sexually transmitted infections (STI) is important for controlling HIV and STI (HIV/STI) among men who have sex with men (MSM). We established multiple machine learning models (e.g., logistic regression, lasso regression, ridge regression, elastic net regression, support vector machine, k-nearest neighbour, naïve bayes, random forest, gradient boosting machine, XGBoost, and multi-layer perceptron) to predict timely (i.e., within 30 days) clinic attendance and HIV/STI testing uptake after receiving a reminder message via short message service (SMS) or email). Our study used 3044 clinic consultations among MSM within 12 months after receiving an email or SMS reminder at the Melbourne Sexual Health Centre between April 11, 2019, and April 30, 2020. About 29.5% [899/3044] were timely clinic attendance post reminder messages, and 84.6% [761/899] had HIV/STI testing. The XGBoost model performed best in predicting timely clinic attendance [mean [SD] AUC 62.8% (3.2%); F1 score 70.8% (1.2%)]. The elastic net regression model performed best in predicting HIV/STI testing within 30 days [AUC 82.7% (6.3%); F1 score 85.3% (1.8%)]. The machine learning approach is helpful in predicting timely clinic attendance and HIV/STI re-testing. Our predictive models could be incorporated into clinic websites to inform sexual health care or follow-up service.

## Introduction

HIV and sexually transmitted infections (STI) have become global public health concerns. Men who have sex with men (MSM) experience a high incidence and prevalence of HIV/STI^1,2^. World Health Organization set a global target to end STI epidemics as public health concerns by 2030^3^. The 2030 agenda for sustainable development called for an end to the AIDS epidemic by 2030^4^. To achieve the UNAIDS 90–90–90 targets for the HIV/AIDS epidemic by 2020 and 95–95–95 targets by 2030, the first “90” or “95” means that 90% of all people living with HIV are aware of their infection status in the world. Globally, about one in four people living with HIV were unaware of their status^5^. Unrecognised HIV/STI due to lack of testing may be one of the driving forces of ongoing HIV/STI transmission among MSM^6^. Delayed HIV diagnosis was also a common problem and caused numerous adverse health consequences^7,8^. Regular attendance enables MSM to access sexual health services and provide timely testing, diagnosis and treatment for HIV and STIs. Improving clinic attendance could also benefit clinical management and long-term follow-up amongst epidemiological priority risk groups. A review demonstrated that having access to health care is essential to control HIV and STIs among MSM^1^.

Short message service (SMS) reminders show a positive impact in improving attendance rates^9^. The widespread use of mobile phones makes it feasible to facilitate sexual health care and behavioural interventions through text messaging (short message service or email). About 81% of the world’s population owned smartphones in 2021^10^, and about 80% of the Australian population used smartphones in 2020^11^. Text message intervention is a viable means to reduce high-risk sexual practices^12^. A systematic review and meta-analysis summarised the effectiveness of text message interventions on prevention, detection, treatment, and knowledge outcomes for STI/HIV^13^. For example, SMS-based interventions could provide tailored health communication messages for MSM living with HIV to provide ongoing health alerts^14^ and have also been used in enhancing antiretroviral therapy adherence in people living with HIV^15,16^.

Machine learning techniques have been used in healthcare^17,18^ but are limited in sexual health services. Machine learning has numerous advantages, such as not requiring statistical inferences and assumptions, identifying complex nonlinear patterns, exploiting complex interactions between risk factors, exploring nonlinear relations among predictors and outcomes, providing accurate prediction, and making predictions at the individual level^19–21^. Despite the advantages of machine learning, little work has been conducted in behavioural modelling using machine learning algorithms for HIV/STIs. To our knowledge, no study used machine learning methods to predict timely clinic attendance and HIV/STI testing among MSM. We only identified one machine-learning study that predicted HIV testing among substance use disorder treatment program participants in the US^22^. We also identified a few machine learning studies predicting sexual practices, such as sexual recidivism^23^ and high-risk condomless anal sex^24^. The current study established machine learning algorithms to predict timely (i.e., within 30 days) clinic attendance and timely HIV/STI testing post clinic reminder messages in MSM. The predictive models are expected to form part of a tool to increase timely clinic attendance and timely HIV/STI testing among MSM in the clinical setting.

## Results

### Characteristics of study data

Our study included 3044 clinic consultations among 1627 MSM after receiving a reminder message via email only (1509 [49.6%]) or SMS only (932 [30.6%]) or both (603 [19.8%]). Their median age was 31.0 years (interquartile range (IQR) 26.8–37.0 years). About 15.5% [472/3044] reported living with HIV. About 19.1% [582/3044] reported STI symptoms on attendance. Of 3044 consultations included in the analysis, 29.5% [899/3044] visited the clinic within 30 days of receiving a reminder. About 84.6% (761/899) had timely HIV/STI testing post reminder messages. Further details were provided in Table 1 and Supplementary Table S1.

**Table 1:** Characteristics of MSM stratified by their timing of clinic attendance after receiving a reminder message and HIV/STI testing post clinic reminder message.

Variables	Clinic attendance		Uptake of HIV/STI testing Within 30 days		Uptake of HIV/STI testing Within 1 year	
	> 30 days (n = 2145)	≤ 30 days (n = 899)	No (n = 138)	Yes (n = 761)	No (n = 347)	Yes (n = 2697)
Age [years, median (IQR)]	33.0 (27.0–39.5)	31.0 (26.0–37.0)	31.0 (27.0–39.8)	30.0 (26.0–37.0)	33.0 (27.0–39.5)	31.0 (26.0–37.0)
**Clinic attendance reminder methods**						
Email and SMS	398 (18.6%)	205 (22.8%)	30 (21.7%)	175 (23.0%)	44 (12.7%)	559 (20.7%)
Email only	1038 (48.4%)	471 (52.4%)	63 (45.7%)	408 (53.6%)	105 (30.3%)	1404 (52.1%)
SMS only	709 (33.1%)	223 (24.8%)	45 (32.6%)	178 (23.4%)	198 (57.1%)	734 (27.2%)
**Screening reminder frequency**						
12 monthly	26 (1.2%)	9 (1.0%)	116 (84.1%)	658 (86.5%)	4 (1.2%)	31 (1.1%)
3 monthly	1754 (81.8%)	774 (86.1%)	22 (15.9%)	94 (12.4%)	271 (78.1%)	2257 (83.7%)
6 monthly	365 (17.0%)	116 (12.9%)	0 (0%)	9 (1.2%)	72 (20.7%)	409 (15.2%)
**Triage reason as asymptomatic screen**						
No	1654 (77.1%)	660 (73.4%)	137 (99.3%)	523 (68.7%)	343 (98.8%)	1971 (73.1%)
Yes	491 (22.9%)	239 (26.6%)	1 (0.7%)	238 (31.3%)	4 (1.2%)	726 (26.9%)
**Triage reason as STI symptoms**						
No	1712 (79.8%)	750 (83.4%)	129 (93.5%)	621 (81.6%)	322 (92.8%)	2140 (79.3%)
Yes	433 (20.2%)	149 (16.6%)	9 (6.5%)	140 (18.4%)	25 (7.2%)	557 (20.7%)
**Triage reason as contact of infection**						
No	1987 (92.6%)	850 (94.5%)	137 (99.3%)	713 (93.7%)	343 (98.8%)	2494 (92.5%)
Yes	158 (7.4%)	49 (5.5%)	1 (0.7%)	48 (6.3%)	4 (1.2%)	203 (7.5%)

*IQR* interquartile range.

### Predictive models for timely clinic attendance post reminder messages

**Figure 1:**
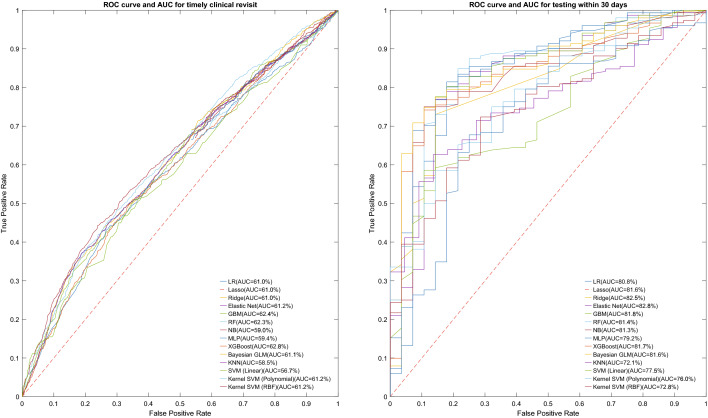
AUC-ROC curves for different machine learning algorithms. (**a**) AUC-ROC curves for different machine learning algorithms on the prediction of clinic attendance within 30 days after receiving a reminder message. (**b**) AUC-ROC curves for different machine learning algorithms on timely HIV/STI testing. *ROC* Receiver operating characteristic, *AUC* area under the ROC curve, *LR* logistic regression, *Lasso* LASSO regression, *Ridge* ridge regression, *Elastic Net* elastic net regression, *GBM* gradient boosting machine, *RF* Random Forest, *NB* Naïve Bayes, *MLP with two hidden layers* two hidden layers multi-layer perceptron neural network, *XGBoost* Extreme Gradient Boosting, *Bayesian GLM* Bayesian Generalized Linear Model, *KNN* K-Nearest Neighbour, *SVM (Linear)* Linear Support Vector Machines (without kernel extensions), *Kernel SVM (Polynomial)* SVM Using Polynomial Basis Kernel, *Kernel SVM (RBF)* SVM Using Radial Basis Function Kernel.

Machine learning algorithms outperform conventional logistic regressions in predicting timely clinic attendance post reminder messages. We found nine machine learning algorithms (XGBoost, gradient boosting machine, random forest, elastic net regression, Kernel SVM (RBF), Bayesian generalised linear model, lasso regression, ridge regression, and logistic regression) with AUC > 60.0% (range 61.0–62.8%). Of the developed machine learning models, the XGBoost model outperformed all other models and provided acceptable performance in predicting timely clinic attendance after receiving the reminder [mean [SD] AUC 62.8% (3.2%); mean [SD] F1 score 70.8% (1.2%)], followed by the gradient boosting machine [AUC 62.4% (1.6%); F1 score 62.4% (0.9%)] (Fig. 1). Details were provided in Supplementary Table S2. The calibration plot for predicting clinic attendance within 30 days using best model (XGBoost) was provided in Supplementary Fig. S1.

**Figure 2:**
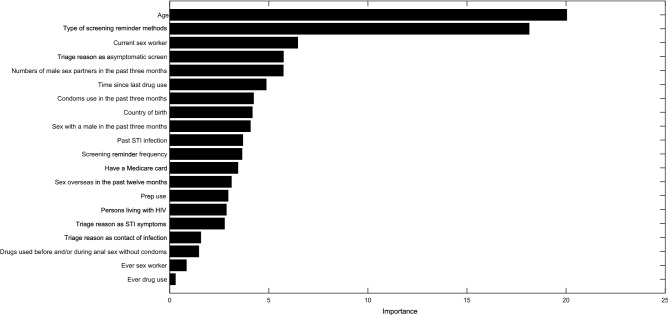
Variable importance in the prediction of timely clinic attendance after receiving a clinic reminder message by XGBoost.

The above section shows that the XGBoost model achieved the best performance. Variable importance analysis using XGBoost identified variables that influenced the prediction of timely clinic attendance post reminder messages (Fig. 2). The contribution rate of the top 10 important predictors for predicting timely clinic attendance post reminder messages were age, type of clinic attendance reminder method, current sex worker, triage reason as asymptomatic screen, numbers of male sex partners in the past three months, time since last drug use, condoms use in the past three months, country of birth, sex with a male in the past three months, and past STI infection. Besides, we also provided the results of variable importance analysis performed using gradient boosting machine, random forest, and elastic net regression in the supplementary materials (Supplementary Figs. S2–4).

### Predictive models for timely HIV/STI testing post reminder messages

We found nine machine learning algorithms (elastic net regression, ridge regression, gradient boosting machine, XGBoost, Bayesian generalised linear model, lasso regression, random forest, naïve bayes, logistic regression) with AUC > 80.0% (range 80.8–82.7%). Of the developed machine learning models, the elastic net regression model outperformed all other models for predicting timely HIV/STI testing [mean [SD] AUC 82.7% (6.3%); mean [SD] F1 score 85.3% (1.8%)], followed by ridge regression [mean [SD] AUC 82.5% (5.4%); mean [SD] F1 score 84.4% (2.4%)]. (Fig. 1; Supplementary Table S3). The calibration plot for predicting HIV/STI testing within 30 days using best model (elastic net regression) was provided in Supplementary Fig. S5. In the supplementary material, we also provided the prediction of HIV/STI testing post clinic reminder messages within 1 year. We found ten machine learning algorithms (XGBoost, elastic net regression, Bayesian generalised linear model, lasso regression, ridge regression, logistic regression, random forest, gradient boosting machine, multi-layer perceptron, and naïve bayes) with AUC > 80.0% (range 80.4–83.7%). The XGBoost algorithm performed best in predicting HIV/STI testing post clinic reminder messages within 1 year [mean [SD] AUC 83.7% (1.8%); mean [SD] F1 score 87.6% (0.8%)] (Supplementary Figs. S6, S7; Supplementary Table S4).

**Figure 3:**
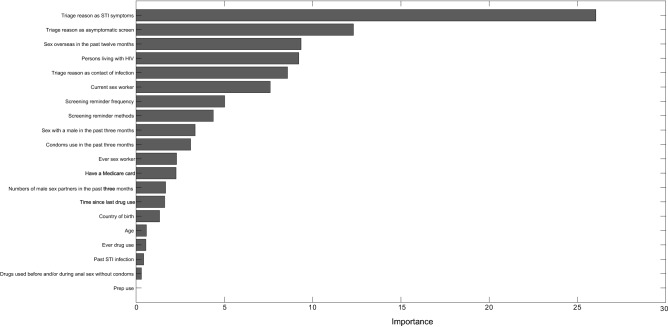
Variable importance in the prediction of timely HIV/STI testing after receiving a clinic reminder message by elastic net regression.

As shown in the above section, the elastic net regression model achieved the best performance. Variable importance analysis using elastic net regression indicated which parameters influenced the prediction of timely HIV/STI testing. (Fig. 3). The top contribution rate of the 10 important predictors for predicting timely HIV/STI testing were triage reasons as STI symptoms, triage reason as asymptomatic screen, sex overseas in the past 12 months, persons living with HIV, triage reason as contact of infection, current sex worker, screening reminder frequency, type of screening reminder method, sex with a male in the past 3 months, and condoms use in the past 3 months. Besides, we also provided the results of variable importance analysis performed using XGBoost, gradient boosting machine, and random forest in the supplementary materials. (Supplementary Figs. S8–10). In the supplemental material, we also provided the variable importance analysis of predicting HIV/STI testing post clinic reminder messages within one year. (Supplementary Fig. S11–14).

## Discussion

About one in three MSM visited the clinic timely (within 30 days) after receiving a reminder message. Among those who visited the clinic timely, about four in five had HIV/STI testing. To our knowledge, this is the first study using machine learning algorithms to predict timely clinic visits and timely HIV/STI testing among MSM post clinic reminder messages. XGBoost showed higher prediction accuracy than the classical multivariate logistic regression model for the prediction of clinic attendance and HIV/STI testing. Our machine learning model provided good performance for predicting timely HIV/STI testing but fair performance in predicting clinic attendance post reminder message. Our findings show that the machine learning-based approach may help predict the use of sexual health services based on existing routinely collected data from electronic health records in clinical settings. Our machine learning models have potential value as a behavioural intervention tool and can be incorporated into clinic websites to inform sexual health care or follow-up service. Variable importance analysis from machine learning models found that the type of screening reminder method was the second most important predictor for predicting clinic attendance after age.

Our results found that the clinic attendance reminder method via email and SMS messages was an important predictor of timely clinic attendance. In addition, our exploratory statistical analysis using multivariable logistic regression found that emailing text messages could be more effective than SMS alone to increase the rate of timely clinic attendance (Supplementary Fig. S15) and timely HIV/STI testing (Supplementary Fig. S16) among MSM. To our knowledge, this is the first comparison of the difference between SMS and email on revisiting the clinic within 30 days or uptake of HIV/STI testing in MSM. Our results suggest the need for further research to investigate the difference between SMS and email on 30-day return visit post clinic reminder message or uptake of HIV/STI testing in different populations. We are also developing an internet-based HIV/STI risk assessment tool. Based on this finding, the clinics could send reminders via email or SMS text messages containing a web link to HIV/STI risk assessment tool to encourage timely return visits and healthcare service seeking.

Our results demonstrate that the machine learning-based approach is useful to predict timely clinic attendance behaviour after receiving a reminder message. The machine learning models can be potentially used to promote timely clinic attendance, which would be helpful to improve the decision-making and initiate pre-emptive interventions in SMS reminders service in sexual health clinics. A previous study found that factors such as STI symptoms, individual attitude and beliefs (e.g., “my partner would blame me if I had an STD”) could cause a delay in seeking care and testing for STI^25^. This study also suggested that those who delayed seeking care at an STI clinic might be interested in home testing^25^. For example, for those individuals who are classified as not having timely clinic attendance, the nurses may remind them multiple times or take further intervention measures (e.g., providing telemedicine services or home testing methods).

The emerging machine learning methods are potentially helpful for healthcare outcomes^26^. Our machine learning models suggest that existing routinely collected data in the electronic health records have some predictive value in predicting sexual health clinic attendance behaviours after receiving the reminder. But our machine learning model performed fairly in predicting 30-day return visits post clinic reminder message. This suggests that other important factors influencing clinic attendance maybe not be included in the routinely collected data. The prediction accuracy of clinic attendance could be further improved by considering additional factors, such as psychological and social characteristics (e.g., greater life satisfaction)^27^. Future machine learning models may include these factors to develop a more accurate predictive model. In this study, we also used machine learning algorithms to assess the effect of the methods of reminders (reminders via SMS or email) on sexual clinic attendance. Our machine learning model also identified some crucial predictors of predicting clinic attendance within 30 days, such as age, current sex workers; this could provide implications for selecting important predictors in future machine learning studies to predict sexual clinic attendance.

Our findings demonstrate that the machine learning models using routine questions in a clinical setting could predict timely HIV/STI testing behaviour. Prediction of timely HIV/STI testing may allow timely intervention measures to increase HIV/STI testing uptake and early diagnosis among the high-risk population. Our machine learning models trained used structured data from electronic health records in clinical settings could be translated into sexual health service products or health intervention tools for medical staff. Our machine learning models provide a powerful potential tool for predicting HIV/STI testing behaviour using routinely collected data in clinical settings. Our models can be potentially used as an HIV/STI testing behavioural intervention tool to promote HIV/STI testing rates among individuals classified as not timely HIV/STI testing. An online survey reported that fear of testing or relying on symptoms of infection were the main reason for not testing among MSM^28^. For example, based on the prediction using routinely collected data using EHRs, medical staff can find the MSM who are classified as not timely HIV/STI testing and provide customised information or reminder (e.g., symptoms do not always appear in the early stage of HIV/STI infection) when seeing the patient or using SMS reminders after seeing a patient to reduce delaying or avoiding HIV/STI testing rates^28^.

This study has some limitations. First, the predictive factors’ validity depends on the accuracy of the self-reported information subjected to participants’ recall and non-response or declined bias. Substantial work has been undertaken on the CASI system’s validity and accuracy^29,30^. Second, some factors might affect timely clinic visits and uptake of HIV/STI testing post clinic reminder messages that have not been included in this study. For example, a review study summarised the barriers to accessing sexual health services, including internalised stigma, confidentiality concerns, sexual health literacy, and fatalism^31^. However, the findings are from male sex workers, and further studies will be needed on the role of these factors. Third, our models’ generalisability to the general MSM population may be limited. Our data were derived from MSM attending a single sexual health service. Our predictive model of HIV/STI testing behaviour is exploratory. Our current study only included MSM who received reminder messages and revisited our clinic. We did not include MSM who received reminder messages and revisited the clinic after one year and those MSM who did not receive reminder messages. There was no information on whether men visited another clinic (e.g., their general practitioner) after receiving reminder messages. We did not record why people who attended within 30 days of the reminder did not get HIV/STI tested. Despite this, our study still provides a meaningful exploration of the application of machine learning in predicting HIV/STI testing. Fifth, we included data up to April 30, 2020, which might introduce selection bias due to the COVID-19 lockdown. The first COVID-19 lockdown in Melbourne started on March 30, 11:59 p.m. and ended on May 12, 11:59 p.m. Our study included a small number of clinic consultations during the COVID-19 lockdown period. Previous studies showed that the re-testing patterns and sexual practices might have changed due to COVID-19, although MSHC remained open during COVID-19 lockdown^32,33^. Six, variable importance analysis did not directly provide the associations' directionality between predictors and outcomes. Seven, we used data from one clinic that services a population with specific demographic characteristics and HIV/STI incidence rates. This may not be representative of patients in other clinics. Therefore, if patients from other clinics are not similar to those attending our clinic service, the predictions from this study may not apply. Further validation is recommended for the prediction model using data from other clinics.

## Methods

### Study design, setting, and participants

#### Short message service reminder programme in Melbourne Sexual Health Centre

Melbourne Sexual Health Centre (MSHC) is Victoria’s largest public sexual health clinic in Australia and offers free HIV/STI testing and management^34^. Men who reported having sex with men in the past 12 months were defined as MSM. Since February 2009, MSHC has offered MSM regular short message service reminders. The content of the email/SMS reminders was: “[Name], your next check-up at MSHC is now due. Please phone 0393416200 for an appointment. To stop reminder, reply 'stop'. To change frequency, reply 'change frequency to' 3, 6, 12 months”.

Our study data included MSM aged 18 years or old who received a reminder message via email or SMS and visited MSHC post clinic reminder messages between April 11, 2019, and April 30, 2020. MSM aged below 18 years were excluded. We excluded men where the clinic attendance post clinic reminder message was longer than 365 days. To test the likely impact of the reminder messages, we excluded clinic attendance after 365 days as it is unlikely that a reminder message from more than one year ago would influence the decision to attend a clinic. Ethics approval was granted by the Alfred Hospital Ethics Committee, Australia (project number: 731/19). All methods were carried out in accordance with relevant guidelines and regulations. As this is a retrospective study involving minimal risk to the privacy of the study subjects, informed consent was waived by the Alfred Hospital Ethics Committee. All identifying details of the study subjects were removed before any computational analysis.

### Main outcome and predictors

The primary study outcome was timely clinic attendance post clinic reminder message. Previous studies used hospital revisits within 30 days^35,36^, so our study considered clinic attendance within 30 days post clinic reminder messages as timely. The time interval between sending the reminder and clinic visit was recorded. The secondary study outcome was the uptake of HIV or STI (gonorrhoea, chlamydia, syphilis) testing (HIV/STI testing) after receiving a reminder message. Timely HIV/STI testing was defined as was the uptake of HIV/STI testing within 30 days post clinic reminder messages.

The screening reminder group was included as a predictor of clinic attendance reminder methods (only email reminders, only SMS reminders, and both email and SMS reminders). And another was the screening reminder frequency, including three-monthly, six-monthly and twelve-monthly.

A literature review and expert discussion informed predictor selection. Socio-demographic factors included age and country of birth. We included predictor questions about socio-demographic factors (e.g., age and country of birth) sexual practices, HIV infection status, past STI diagnosis, reasons for attendance (e.g., contact of infection, symptom status), drug use, any sex overseas, participating in sex work, and pre-exposure prophylaxis (PrEP) use. Details in Table 1 and Supplementary Table S1.

### Machine-learning approaches and statistical analysis

We established a series of machine learning models in this study. The choice of these models is arbitrary; however, these models are commonly utilised machine learning models in health care^17,18^, including Logistic Regression (LR), LASSO Regression (Lasso), Ridge Regression (Ridge), Elastic Net Regression (Elastic Net), Gradient Boosting Machine (GBM), Random Forest (RF), Extreme Gradient Boosting (XGBoost), Bayesian Generalized Linear Model (Bayesian GLM), K-Nearest Neighbour (KNN), Linear Support Vector Machines (without kernel extensions) [SVM(Linear)], SVM Using Polynomial Basis Kernel [Kernel SVM (Polynomial)], SVM Using Radial Basis Function Kernel [Kernel SVM (RBF)], Naïve Bayes (NB), and multi-layer feedforward artificial neural networks (MLP).

We conducted random-forest-based imputation to deal with the missing data^37^. The random-forest-based imputation was built using the *mice* package. Our machine learning models used a one-hot encoding scheme for training. To address the selection bias caused by using a single data set for model selection and training, we used a 3 × 10 (3 outer folds, 10 inner folds) nested cross-validation (CV). We used the CV and grid search technique to evaluate and optimise the model hyperparameters to avoid overfitting and improve generalizability. The model and hyperparameters with the largest AUC in internal tenfold cross-validation and grid search were selected as the training outer-loop model and tested on the threefold outer-loop test fold^38,39^.

The performance of machine learning models was evaluated by computing model evaluation indicators, including area under the curve (AUC) and F1 score^40^. The average results of the AUC and F1 score of the 3 outer folds were generated to the performance metrics. The F1 score combines precision (positive predictive value) and recall (sensitivity) and is the harmonic average of precision and recall^41^. The value of the AUC and F1 score is between 0 and 1. A higher value of the AUC or F1-score indicates a better model performance. We also conducted variable importance analysis to reveal the relative importance of predictors in predicting timely clinic attendance and uptake of HIV/ STI testing post reminder message.

LR, Lasso, RidgeR, Elastic Net, GBM, RF, NB, and MLP were built using the *h2o* package. XGBoost was built using the *xgboost* package. Bayesian GLM was built using the *arm* package. KNN was built using the *kknn* package. SVM(Linear) was built using the *e1071* package. Kernel SVM (Polynomial) and Kernel SVM (RBF) were built using the *kernlab* package.

Characteristics of individuals were summarised and presented with descriptive statistics. Multivariable stepwise logistic regression with backwards elimination was used to identify important independent predictors of timely clinic attendance and timely HIV/STI testing uptake. Statistical significance was considered at p < 0.05. Statistical analysis and machine learning algorithms were conducted with R 4.0.3. We used MATLAB R 2019a to plot figures.

## Supplementary Information

Supplementary Information.

## Data Availability

The data is not publicly available due to privacy or ethical restrictions, but will be made available on reasonable request from the corresponding author, with the permission of the Alfred Hospital Ethics Committee. Restrictions apply to the availability of these data, which were used under license for this study. The code developed for this study is available on reasonable request from the corresponding author.
