# Supplementary material for: Using machine learning approaches to predict timely clinic attendance and the uptake of HIV/STI testing post clinic reminder messages
Source: Sci Rep. 2022 May 24;12:8757. doi: 10.1038/s41598-022-12033-7 (PMC9128330; doi:10.1038/s41598-022-12033-7)
Supplement: Supplementary file 1 — Supplementary Information. [file 41598_2022_12033_MOESM1_ESM.pdf]

Using machine learning approaches to predict timely clinic attendance and the uptake of HIV/STI testing post clinic reminder messages

Authors: Xianglong Xu[1,2], Christopher K. Fairley[1,2], Eric P.F. Chow[1,2,3], David Lee[2], Ei T Aung[1,2], Lei Zhang[1,2,4,5*], Jason J. Ong[1,2,6*]

[1] Central Clinical School, Monash University, Melbourne, Australia

[2] Melbourne Sexual Health Centre, The Alfred, Melbourne, Australia

[3] Centre for Epidemiology and Biostatistics, Melbourne School of Population and Global Health, The University of Melbourne, Melbourne, Victoria, Australia

[4] China Australia Joint Research Center for Infectious Diseases, School of Public Health, Xi'an Jiaotong University Health Science Centre, Xi'an, Shaanxi, People's Republic of China.

[5] Department of Epidemiology and Biostatistics, College of Public Health, Zhengzhou University, Zhengzhou, China

[6] Faculty of Infectious and Tropical Diseases, London School of Hygiene and Tropical Medicine, London, United Kingdom

*** Authors contribute to supervision equally**

Corresponding author.

- Lei Zhang, China Australia Joint Research Center for Infectious Diseases, School of Public Health, Xi'an Jiaotong University Health Science Centre, Xi'an, Shaanxi, People's Republic of China. Lei.zhang1@monash.edu; +613 9341 6264 (telephone, and fax numbers).

- Jason J. Ong, Melbourne Sexual Health Centre, The Alfred, Melbourne, 3053, Australia. Jason.ong@monash.edu (e-mail); 0401 660 753 (telephone, and fax numbers).

Characteristics of study data

Supplementary Table S1. Characteristics of MSM stratified by their timing of clinic attendance after receiving a reminder message and HIV/STI testing post clinic reminder message

**Variables**	**Clinic attendance(N=3,044)**		**Uptake of HIV/STI testing within one year (N=3,044)**		**Uptake of HIV/STI testing within 30 days (N=899)**	
**HIV status**	**> 30 days (n=2145)**	**<= 30 days (n=899)**	**No (n=138)**	**Yes (n=761)**	**No (n=347)**	**Yes (n=2697)**
HIV-	1825 (85.1%)	747 (83.1%)	76 (55.1%)	671 (88.2%)	202 (58.2%)	2370 (87.9%)
HIV+	320 (14.9%)	152 (16.9%)	62 (44.9%)	90 (11.8%)	145 (41.8%)	327 (12.1%)
**Past STI infection**						
No	1355 (63.2%)	674 (75.0%)	125 (90.6%)	549 (72.1%)	311 (89.6%)	1718 (63.7%)
Yes	790 (36.8%)	225 (25.0%)	13 (9.4%)	212 (27.9%)	36 (10.4%)	979 (36.3%)

**Sex with a male in the past three months**						
No	244 (11.4%)	125 (13.9%)	29 (21.0%)	96 (12.6%)	66 (19.0%)	303 (11.2%)
Yes	1901 (88.6%)	774 (86.1%)	109 (79.0%)	665 (87.4%)	281 (81.0%)	2394 (88.8%)
**Numbers of male sex partners in the past 3 months (Median (IQR))**	0.0(0.0-0.0)	1.0(0.0-4.0)	0.0(0.0-0.0)	1.0(0.0-3.0)	0.0(0.0-0.0)	1.0(0.0-4.0)
**Condoms use in the past three months**						
Always or Usually (>50%)	579 (27.0%)	277 (30.8%)	49 (35.5%)	228 (30.0%)	106 (30.5%)	750 (27.8%)
Never	408 (19.0%)	168 (18.7%)	22 (15.9%)	146 (19.2%)	69 (19.9%)	507 (18.8%)
Sometimes	1158 (54.0%)	454 (50.5%)	67 (48.6%)	387 (50.9%)	172 (49.6%)	1440 (53.4%)
**Ever sex worker**						
No	2063 (96.2%)	884 (98.3%)	138 (100%)	746 (98.0%)	333 (96.0%)	2614 (96.9%)

Yes	82 (3.8%)	15 (1.7%)	0 (0%)	15 (2.0%)	14 (4.0%)	83 (3.1%)
**Current sex worker**						
No	2135 (99.5%)	897 (99.8%)	138 (100%)	759 (99.7%)	347 (100%)	2685 (99.6%)
Yes	10 (0.5%)	2 (0.2%)	0 (0%)	2 (0.3%)	0 (0%)	12 (0.4%)
**Ever drug use**						
No	28 (1.3%)	4 (0.4%)	2 (1.4%)	2 (0.3%)	10 (2.9%)	22 (0.8%)
Yes	2117 (98.7%)	895 (99.6%)	136 (98.6%)	759 (99.7%)	337 (97.1%)	2675 (99.2%)
**Time since last drug use**						
Less than 12 months ago	7 (0.3%)	6 (0.7%)	2 (1.4%)	5 (0.7%)	1 (0.3%)	12 (0.4%)
Less than 3 months ago	28 (1.3%)	7 (0.8%)	136 (98.6%)	743 (97.6%)	5 (1.4%)	30 (1.1%)
More than 12 months ago	19 (0.9%)	7 (0.8%)	0 (0%)	6 (0.8%)	2 (0.6%)	24 (0.9%)
Never injected	2091 (97.5%)	879 (97.8%)	0 (0%)	7 (0.9%)	339 (97.7%)	2631 (97.6%)
**Pre-exposure prophylaxis use**						

No	1600 (74.6%)	656 (73.0%)	113 (81.9%)	543 (71.4%)	268 (77.2%)	1988 (73.7%)
Yes	545 (25.4%)	243 (27.0%)	25 (18.1%)	218 (28.6%)	79 (22.8%)	709 (26.3%)
**Had sex with someone outside (or from outside) Australia or New Zealand in the past twelve months**						
No	1510 (70.4%)	665 (74.0%)	116 (84.1%)	549 (72.1%)	293 (84.4%)	1882 (69.8%)
Yes	635 (29.6%)	234 (26.0%)	22 (15.9%)	212 (27.9%)	54 (15.6%)	815 (30.2%)
**Country of birth**						
Australia	939 (43.8%)	345 (38.4%)	47 (34.1%)	298 (39.2%)	147 (42.4%)	1137 (42.2%)
not Australia	1206 (56.2%)	554 (61.6%)	91 (65.9%)	463 (60.8%)	200 (57.6%)	1560 (57.8%)
**Drugs used before and/or during anal sex without condoms**						

No	1445 (67.4%)	635 (70.6%)	105 (76.1%)	530 (69.6%)	240 (69.2%)	1840 (68.2%)
Yes	700 (32.6%)	264 (29.4%)	33 (23.9%)	231 (30.4%)	107 (30.8%)	857 (31.8%)
**Medicare Card**						
No	573 (26.7%)	308 (34.3%)	47 (34.1%)	261 (34.3%)	82 (23.6%)	799 (29.6%)
Yes	1572 (73.3%)	591 (65.7%)	91 (65.9%)	500 (65.7%)	265 (76.4%)	1898 (70.4%)
**HIVSTI testing**						
No	209 (9.7%)	138 (15.4%)	-	-	-	-
Yes	1936 (90.3%)	761 (84.6%)	-	-	-	-

Note: IQR: interquartile range.

Timely clinic attendance post reminder messages

Supplementary Table S2. Model evaluation of clinic attendance within 30 days on the testing dataset (N=3,044)

Models	Clinic attendance within 30 days (N=3,044)		Clinic attendance within 30 days (N=3,044)	
	AUC (mean/ SD)		F1 score (mean/ SD)	
LR	61.0%	1.8%	54.1%	5.4%
Lasso	61.0%	1.8%	54.3%	5.2%
Ridge	61.0%	1.8%	54.1%	5.4%
Elastic Net	61.2%	2.3%	53.8%	3.4%
GBM	62.4%	1.6%	62.4%	0.9%
RF	62.3%	2.0%	50.3%	6.7%
NB	59.0%	2.8%	46.6%	1.2%
MLP with two hidden layers	59.4%	4.5%	57.4%	5.3%
XGBoost	62.8%	3.2%	70.8%	1.2%
Bayesian GLM	61.1%	1.8%	70.0%	1.1%
KNN	49.8%	0.3%	70.0%	2.7%

SVM(Linear)	58.5%	3.0%	69.4%	2.4%
Kernel SVM (Polynomial)	56.7%	2.2%	70.5%	2.0%
Kernel SVM (RBF)	61.2%	3.2%	70.0%	1.9%

LR= Logistic Regression, Lasso= LASSO Regression, Ridge= Ridge Regression, Elastic Net = Elastic Net Regression, GBM=Gradient Boosting Machine, RF= Random Forest, NB= Naïve Bayes, MLP with two hidden layers = two hidden layers feedforward artificial neural networks, XGBoost = Extreme Gradient Boosting, Bayesian GLM = Bayesian Generalized Linear Model, KNN = K-Nearest Neighbour, SVM (Linear) = Linear Support Vector Machines (without kernel extensions), Kernel SVM (Polynomial) = SVM Using Polynomial Basis Kernel, Kernel SVM (RBF) = SVM Using Radial Basis Function Kernel.

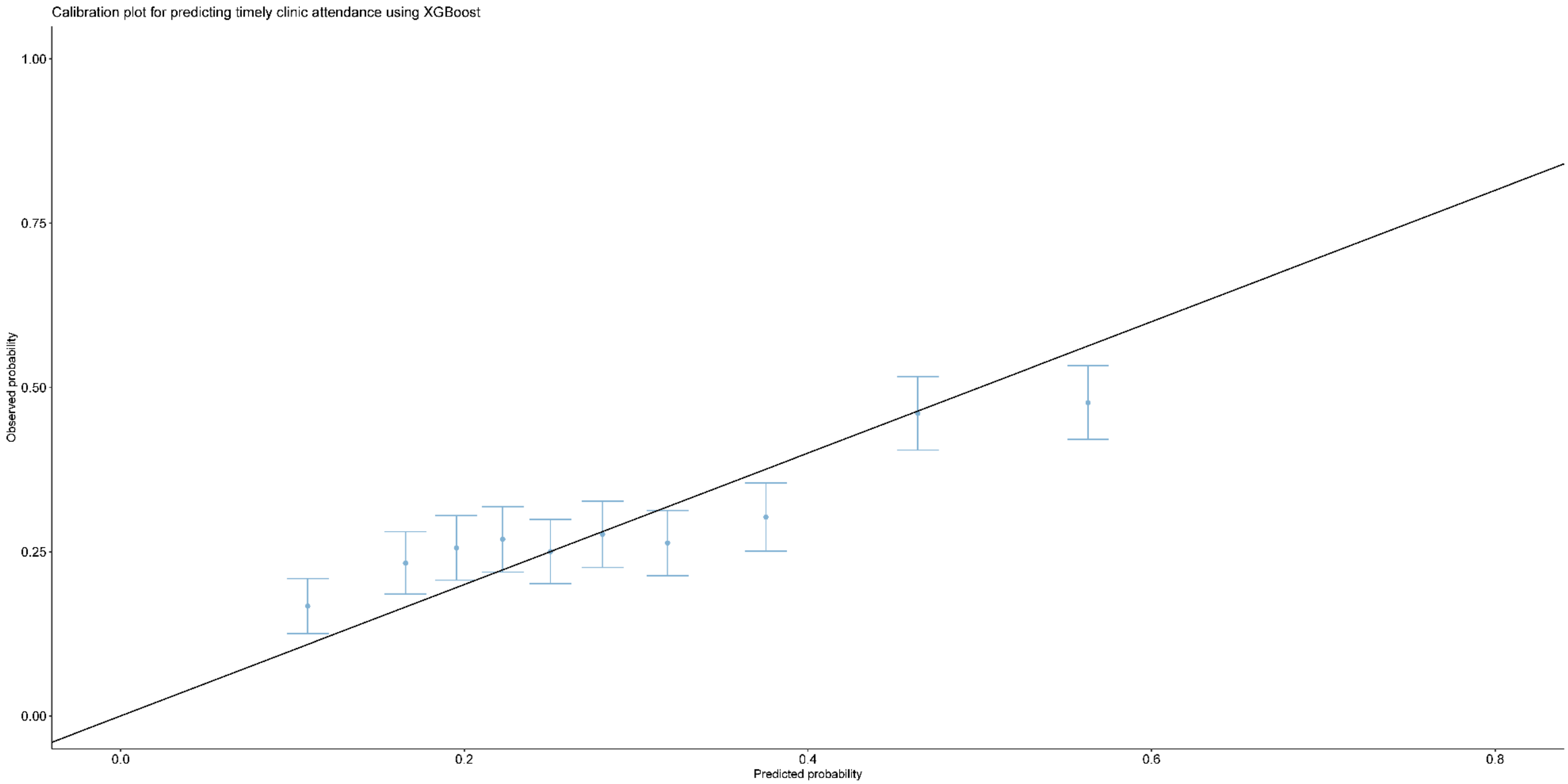

Supplementary Figure S1. Calibration plot for predicting clinic attendance within 30 days using best model (XGBoost) on the testing dataset.

Variable importance analysis

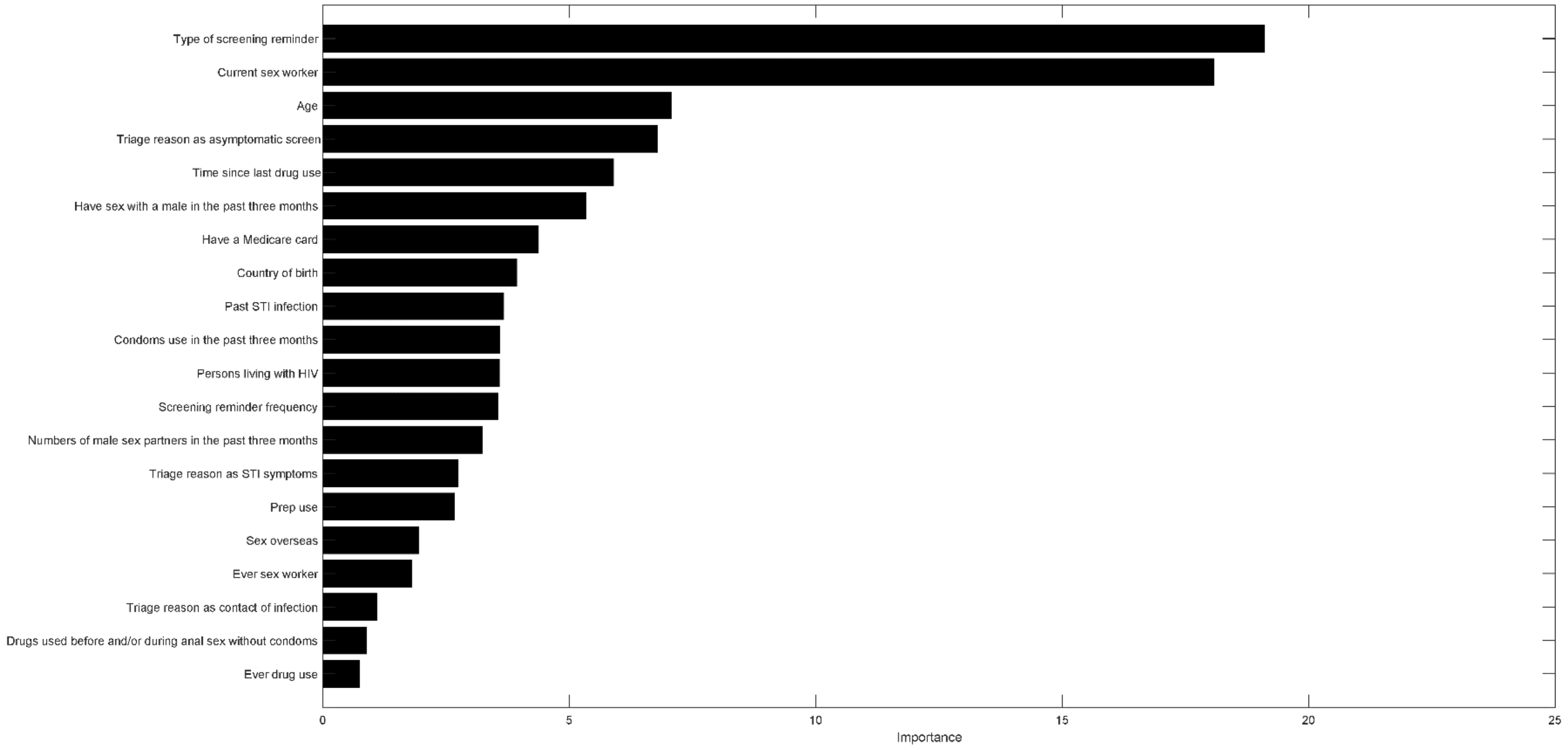

Supplementary Figure S2. Variable importance in the prediction of timely clinic attendance after receiving a reminder message by random forest

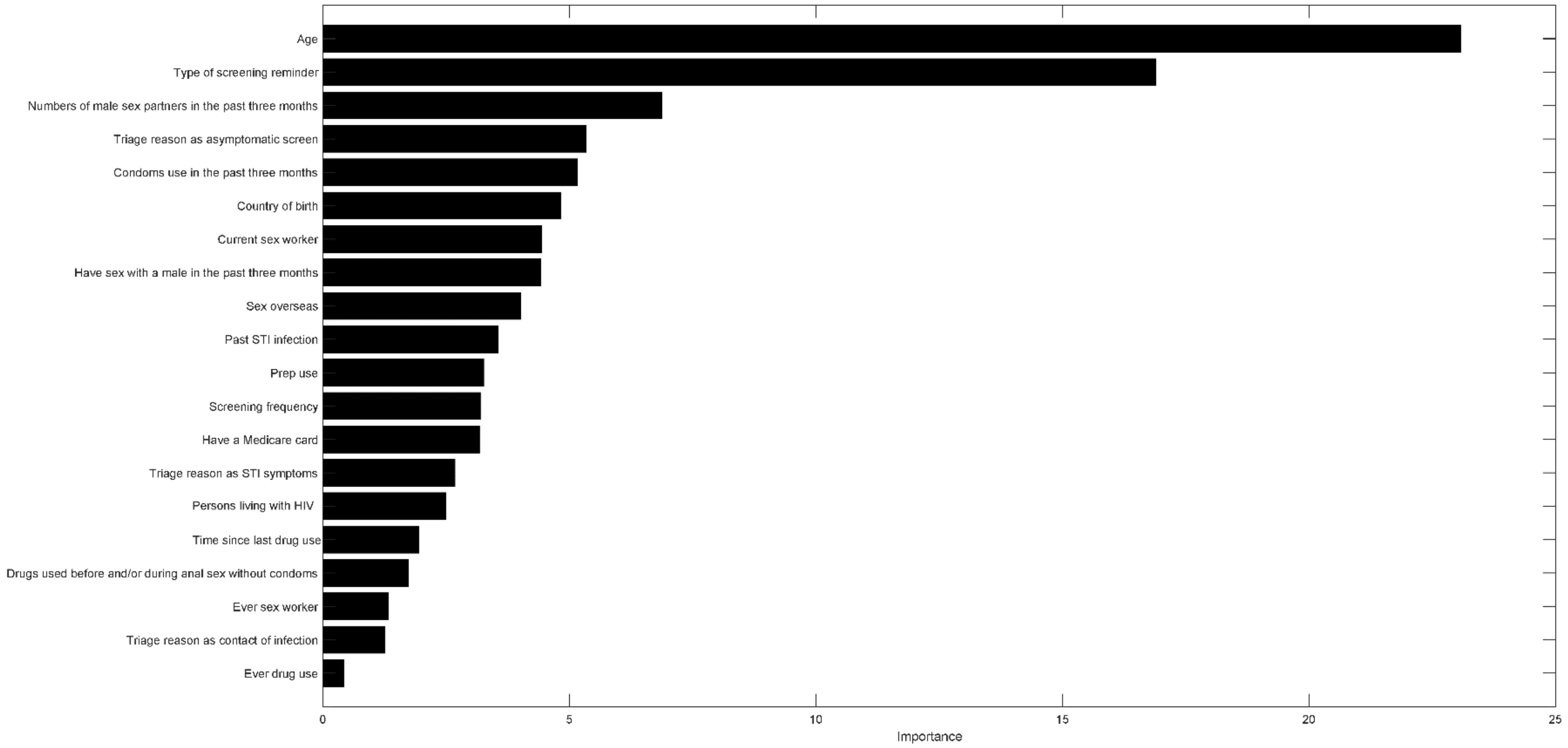

Supplementary Figure S3. Variable importance in the prediction of timely clinic attendance after receiving a reminder message by GBM

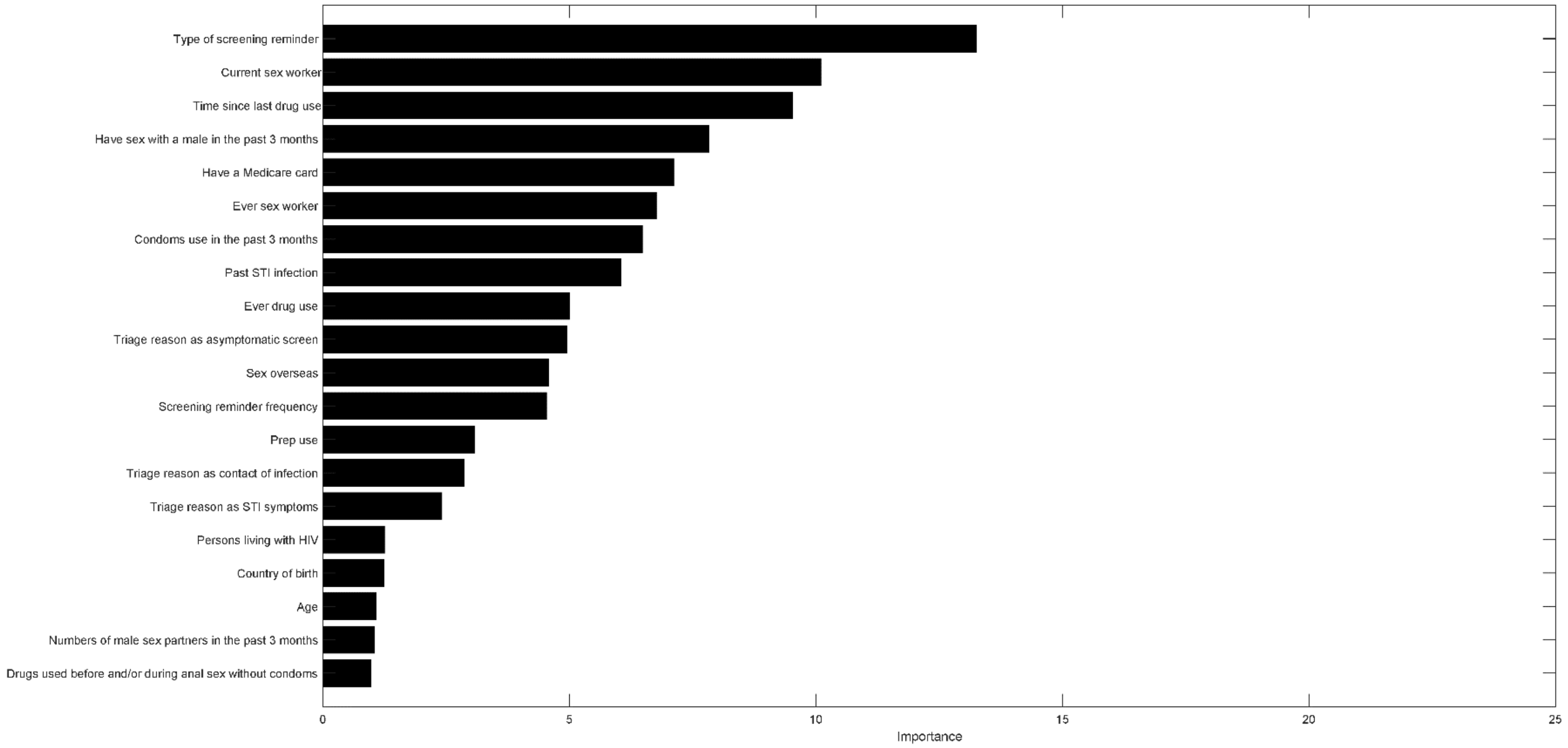

Supplementary Figure S4. Variable importance in the prediction of timely clinic attendance after receiving a reminder message by elastic net regression

HIV/STI testing

HIV/STI testing within 30 days

Supplementary Table S3. Model evaluation of HIV/STI testing within 30 days on the testing dataset (N=899)

Models	HIV/STI testing (N=899)		HIV/STI testing (N=899)	
	AUC (mean/ SD)		F1 score (mean/ SD)	
LR	80.8%	4.1%	84.0%	2.1%
Lasso	81.6%	6.2%	84.6%	1.9%
Ridge	82.5%	5.4%	84.4%	2.4%
Elastic Net	82.7%	6.3%	85.3%	1.8%
GBM	81.8%	5.5%	83.3%	2.7%
RF	81.4%	6.2%	85.5%	1.5%
NB	81.3%	5.6%	73.7%	4.8%
MLP with two hidden layers	79.2%	4.1%	84.0%	0.7%
XGBoost	81.7%	4.3%	83.5%	2.6%
Bayesian GLM	81.6%	5.3%	84.6%	2.1%

KNN	77.9%	2.4%	81.8%	2.7%
SVM(Linear)	72.1%	1.5%	82.9%	1.8%
Kernel SVM (Polynomial)	77.5%	4.4%	85.5%	2.0%
Kernel SVM (RBF)	76.0%	2.6%	85.3%	2.1%

LR= Logistic Regression, Lasso= LASSO Regression, Ridge= Ridge Regression, Elastic Net = Elastic Net Regression, GBM=Gradient Boosting Machine, RF= Random Forest, NB= Naïve Bayes, MLP with two hidden layers = two hidden layers feedforward artificial neural networks , XGBoost = Extreme Gradient Boosting, Bayesian GLM = Bayesian Generalized Linear Model, KNN = K-Nearest Neighbour, SVM (Linear) = Linear Support Vector Machines (without kernel extensions), Kernel SVM (Polynomial) = SVM Using Polynomial Basis Kernel, Kernel SVM (RBF) = SVM Using Radial Basis Function Kernel.

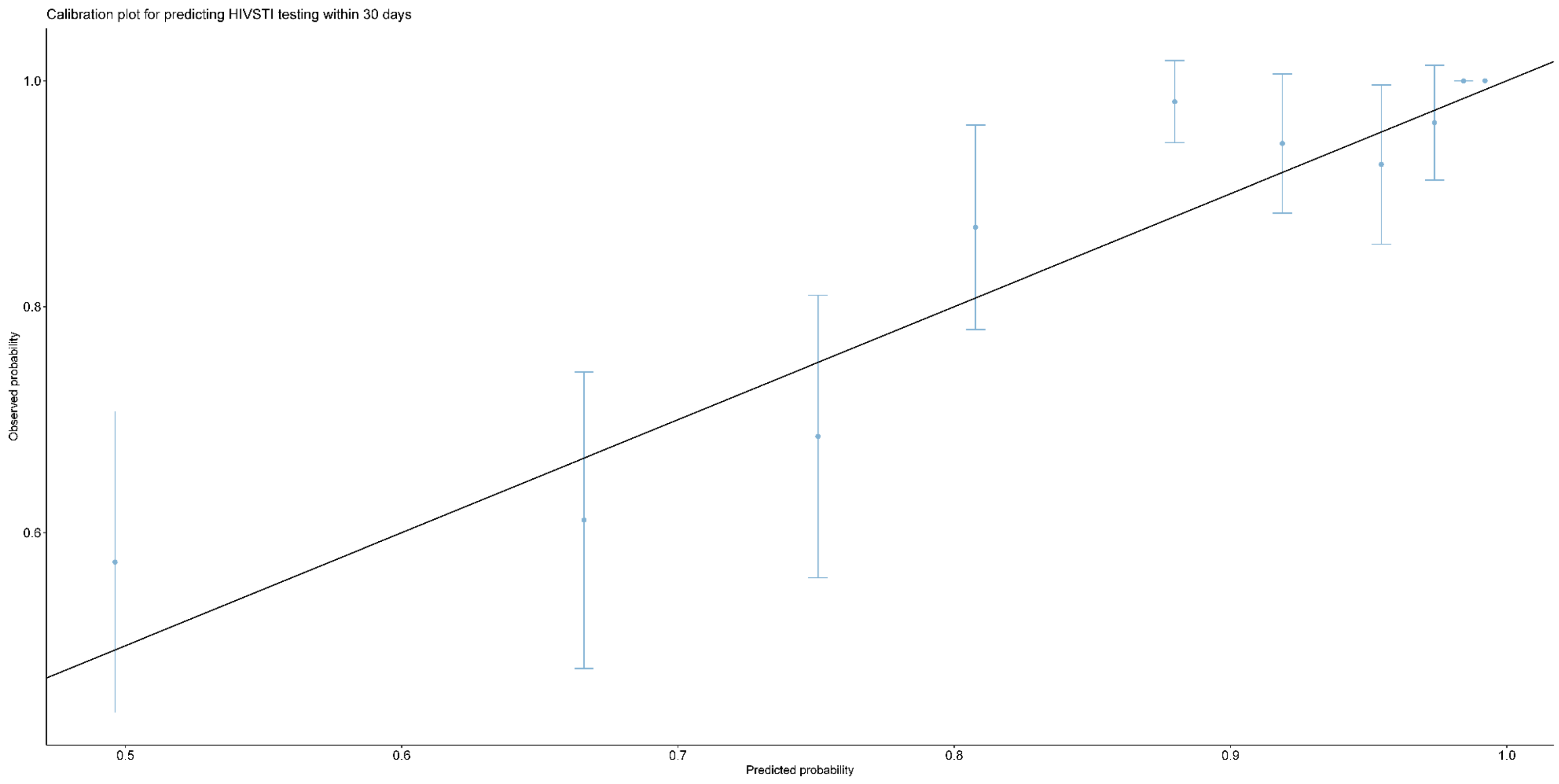

Supplementary Figure S5. Calibration plot for predicting HIV/STI testing within 30 days using best model (elastic net regression) on the testing dataset.

HIV/STI testing within 1-year post reminder message

Machine learning algorithms outperform conventional logistic regressions in predicting HIV/STI testing post clinic reminder messages within one year. Of the 14 machine learning models, the XGBoost model outperformed all other models and provided excellent performance for predicting HIV/STI testing messages within one year (AUC= 0.837; F1 score=0.876), followed by the Bayesian GLM model (AUC= 0.836; F1 score=0.879).

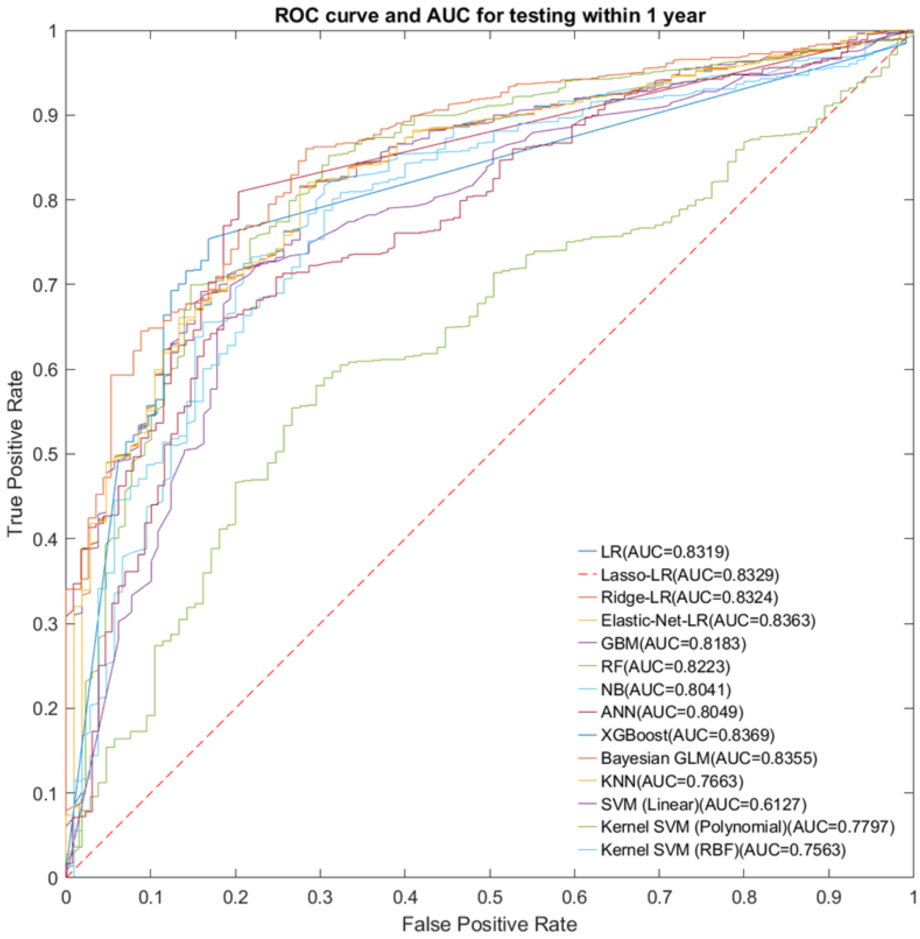

ROC curve and AUC for testing within 1 year
True Positive Rate
False Positive Rate
0
0.1
0.2
0.3
0.4
0.5
0.6
0.7
0.8
0.9
1
LR(AUC=0.8319)
Lasso-LR(AUC=0.8329)
Ridge-LR(AUC=0.8324)
Elastic-Net-LR(AUC=0.8363)
GBM(AUC=0.8183)
RF(AUC=0.8223)
NB(AUC=0.8041)
ANN(AUC=0.8049)
XGBoost(AUC=0.8369)
Bayesian GLM(AUC=0.8355)
KNN(AUC=0.7663)
SVM (Linear)(AUC=0.6127)
Kernel SVM (Polynomial)(AUC=0.7797)
Kernel SVM (RBF)(AUC=0.7563)

Supplementary Figure S6. The receiver operating characteristic (ROC) curve and area under the curve (AUC) for clinical testing within 1 year.

LR= Logistic Regression, Lasso-LR= LASSO Regression, Ridge-LR= Ridge Regression, Elastic-Net-LR= Elastic-Net Regression, GBM=Gradient Boosting Machine, RF= Random Forest, NB= Naïve Bayes, MLP = two hidden layers feedforward artificial neural networks XGBoost = Extreme Gradient Boosting, Bayesian GLM = Bayesian Generalized Linear Model, KNN = K-Nearest Neighbour, SVM (Linear) = Linear Support Vector Machines (without kernel extensions), Kernel SVM (Polynomial) = SVM Using Polynomial Basis Kernel, Kernel SVM (RBF) = SVM Using Radial Basis Function Kernel.

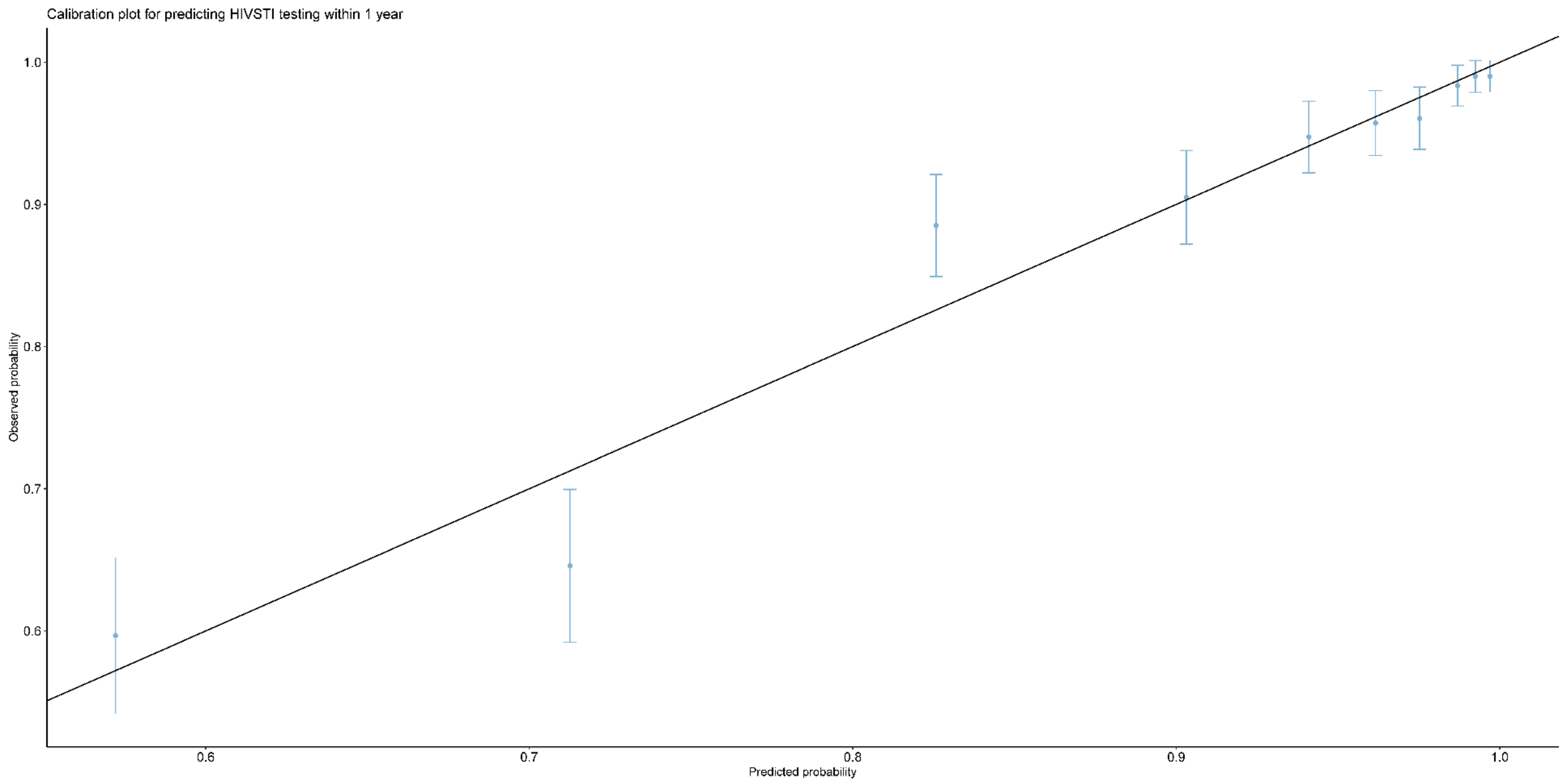

Supplementary Figure S7. Calibration plot for predicting HIV/STI testing within 1-year using best model (XGBoost) on the testing dataset.

Supplementary Table S4. Model evaluation of HIV/STI testing within one year on the testing data set (N=3,044)

Models	HIV/STI testing (N=899)	HIV/STI testing (N=899)

	AUC (mean/ SD)		F1 score (mean/ SD)	
LR	83.2%	2.1%	87.9%	0.9%
Lasso	83.3%	2.0%	87.9%	0.9%
Ridge	83.2%	2.0%	87.9%	0.9%
Elastic Net	83.6%	1.8%	88.3%	1.0%
GBM	81.8%	1.9%	87.2%	0.7%
RF	82.2%	2.5%	88.5%	1.1%
NB	80.4%	3.3%	85.5%	5.1%
MLP with two hidden layers	80.5%	1.5%	88.3%	0.8%
XGBoost	83.7%	1.8%	87.6%	0.8%
Bayesian GLM	83.6%	1.7%	87.9%	1.6%
KNN	71.7%	4.0%	87.1%	1.1%
SVM(Linear)	76.6%	0.3%	87.9%	0.5%
Kernel SVM (Polynomial)	61.3%	10.5%	88.6%	1.2%
Kernel SVM (RBF)	78.0%	1.9%	88.3%	1.1%

LR= Logistic Regression, Lasso= LASSO Regression, Ridge= Ridge Regression, Elastic Net = Elastic Net Regression, GBM=Gradient Boosting Machine, RF= Random Forest, NB= Naïve Bayes, MLP with two hidden layers XGBoost = Extreme Gradient Boosting, Bayesian GLM = Bayesian

Generalized Linear Model, KNN = K-Nearest Neighbour, SVM (Linear) = Linear Support Vector Machines (without kernel extensions), Kernel SVM (Polynomial) = SVM Using Polynomial Basis Kernel, Kernel SVM (RBF) = SVM Using Radial Basis Function Kernel.

Variable importance analysis

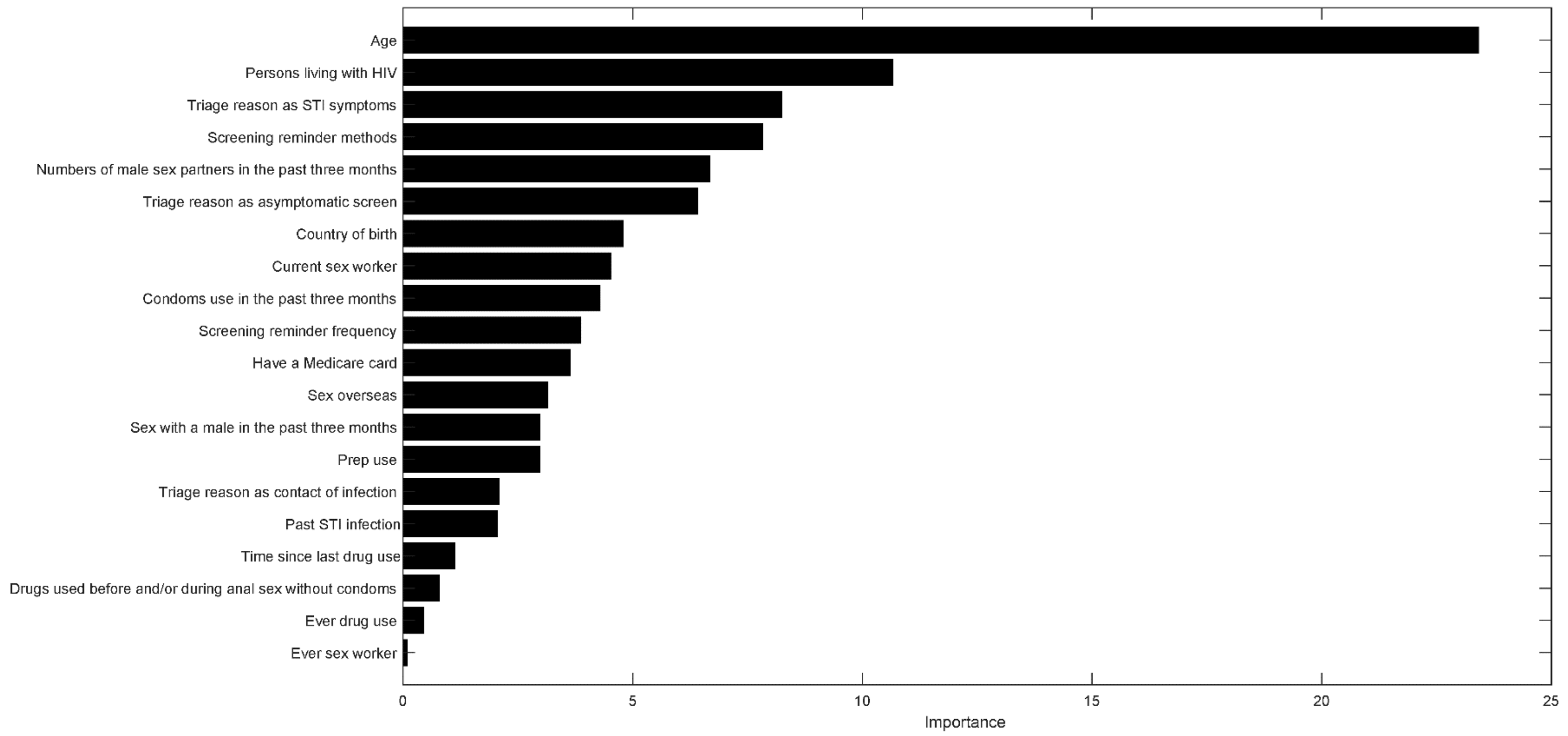

Supplementary Figure S8. Variable importance in the prediction of timely HIV/STI testing after receiving a reminder message by XGBoost

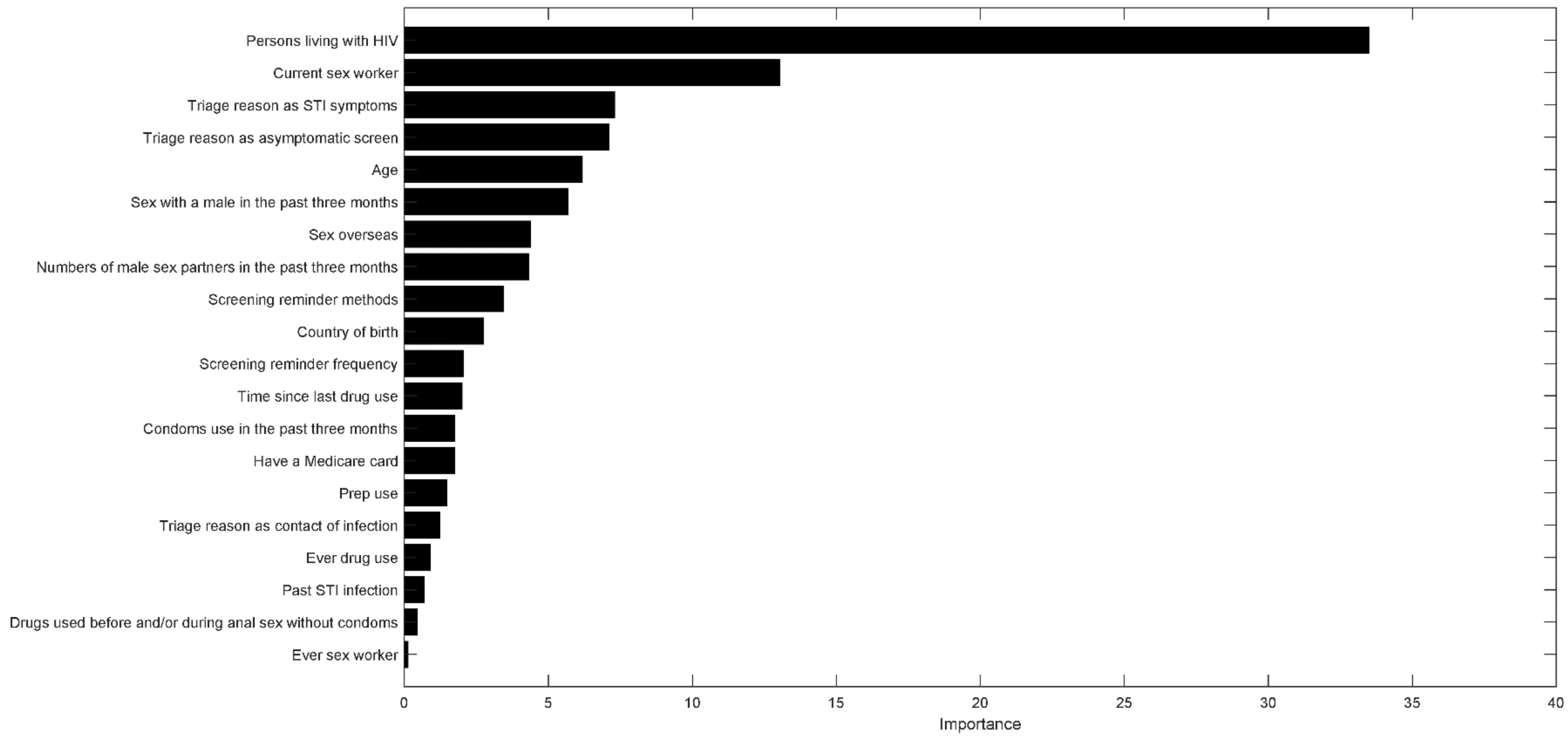

Supplementary Figure S9. Variable importance in the prediction of timely HIV/STI testing after receiving a reminder message by random forest

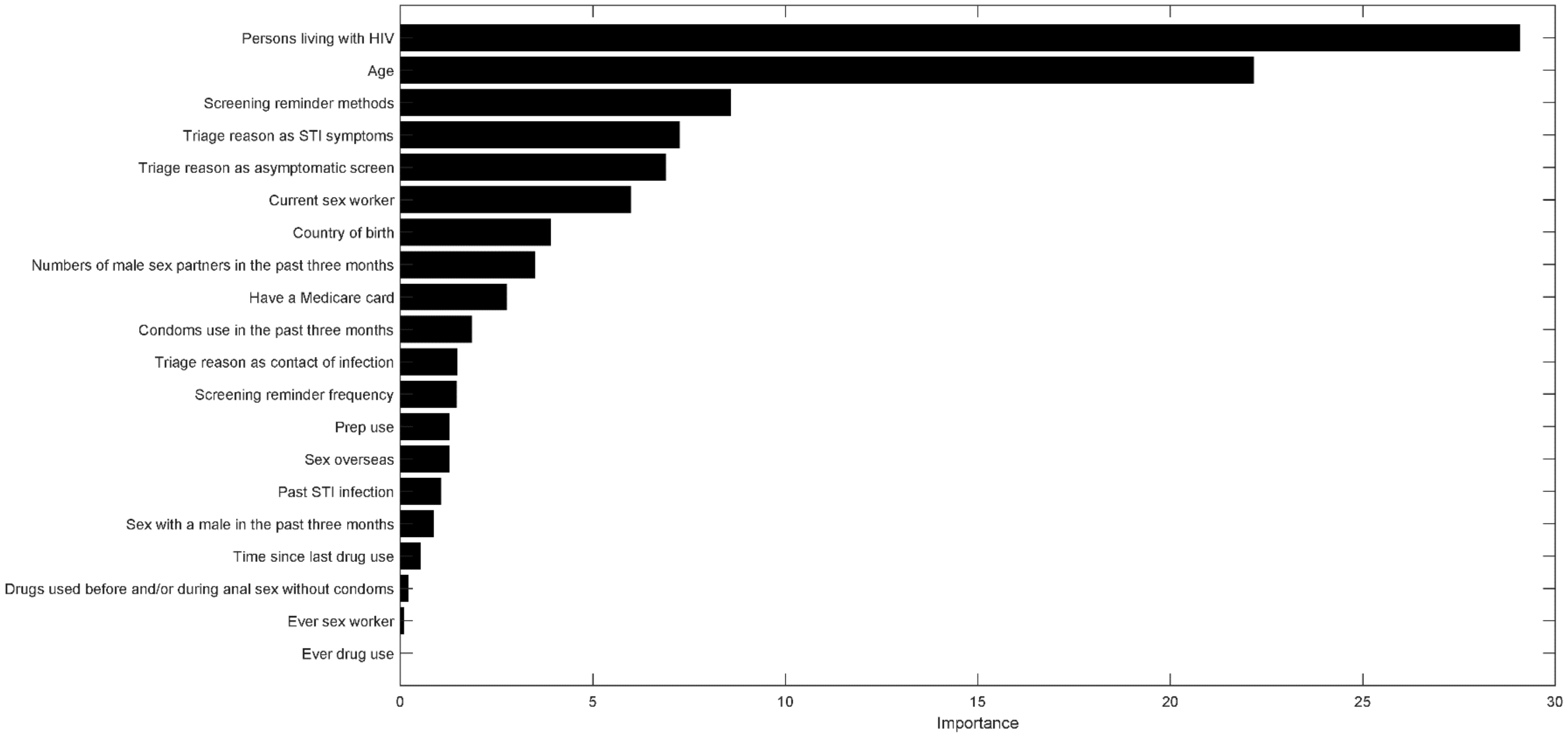

Supplementary Figure S10. Variable importance in the prediction of timely HIV/STI testing after receiving a reminder message by GBM

Variable importance analysis using XGBoost indicated which parameters influenced the prediction of HIV/STI testing within 1 year. The top 10 important predictors for predicting uptake of HIV/STI testing post clinic reminder messages within one year were age, clinic attendance reminder methods, triage reason as asymptomatic screen, sex with a male in the past three months, numbers of male sex partners in the past three months, triage reason as symptomatic, persons living with HIV, current sex worker, clinic attendance within 30 days, and condom use in the past three months.

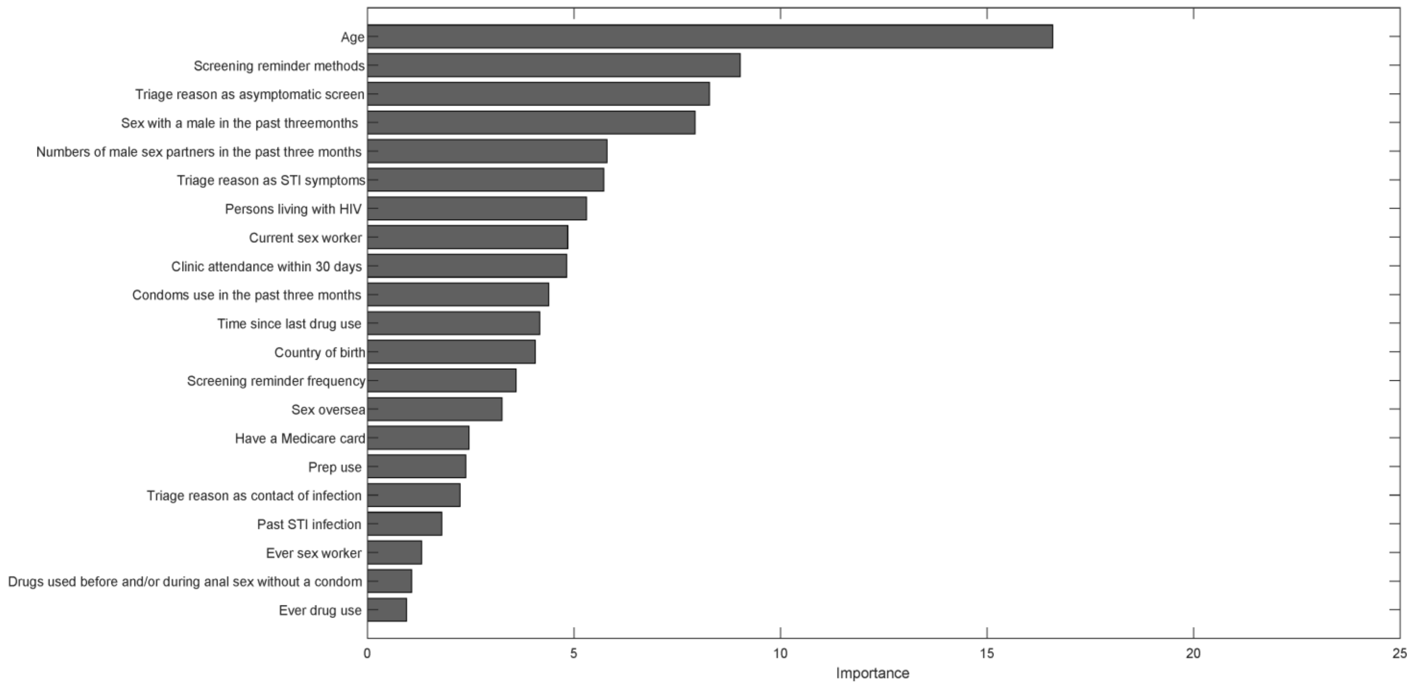

Supplementary Figure S11. Variable importance in the prediction of HIV/STI testing post clinic reminder message within one year by XGBoost

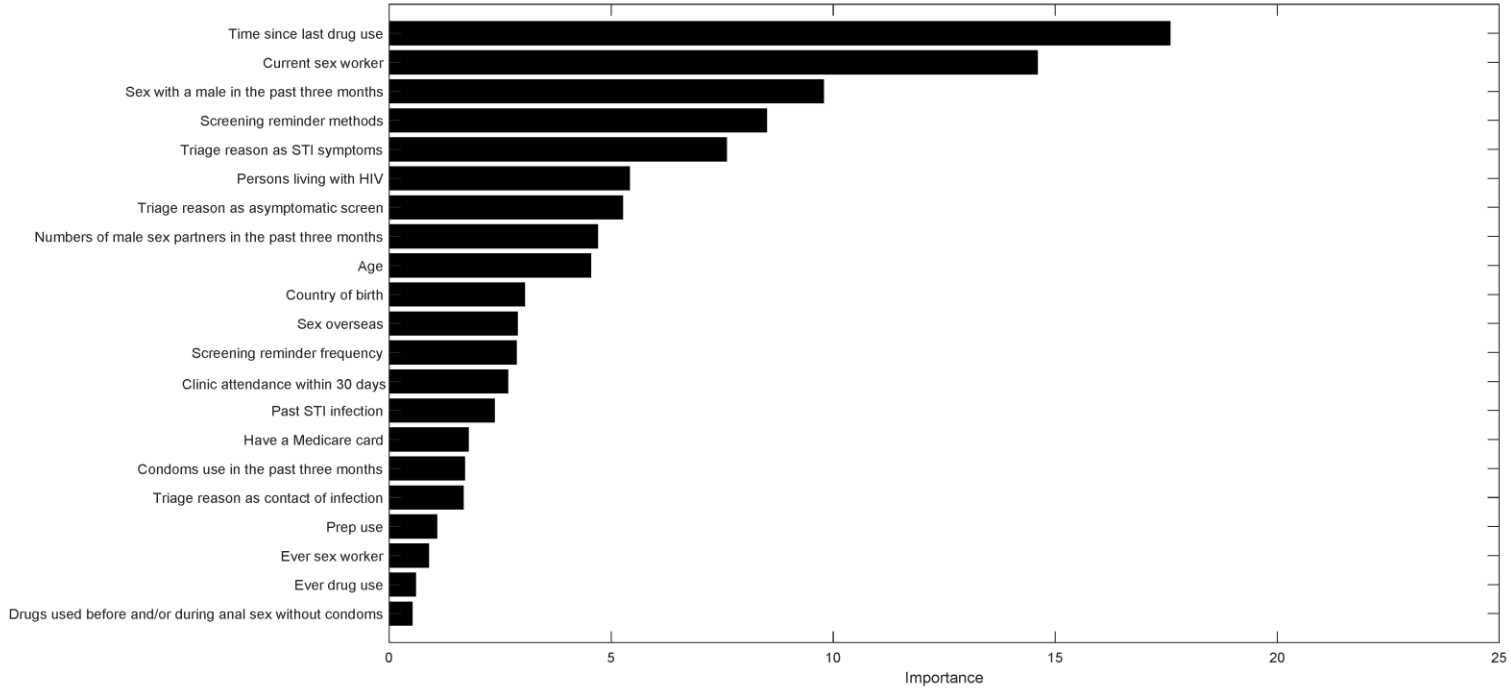

Supplementary Figure S12. Variable importance in the prediction of HIV/STI testing post clinic reminder message within one year by random forest

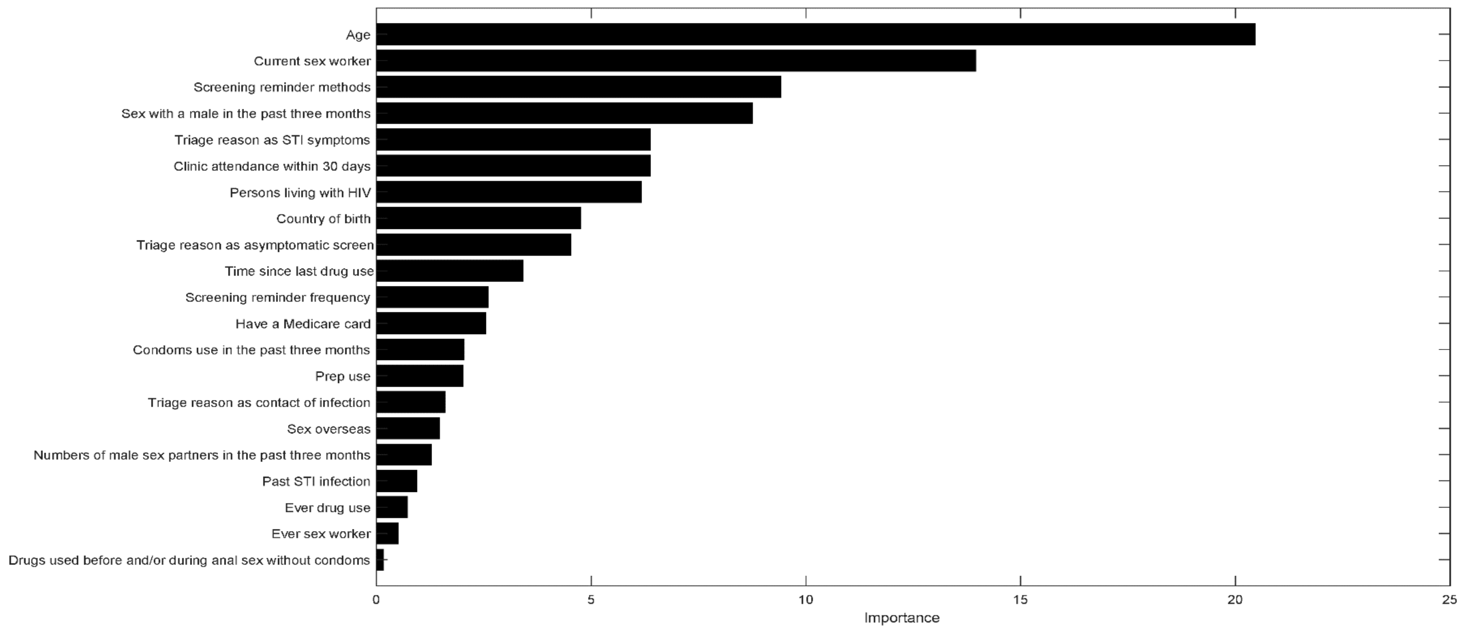

Supplementary Figure S13. Variable importance in the prediction of HIV/STI testing post clinic reminder message within one year by GBM

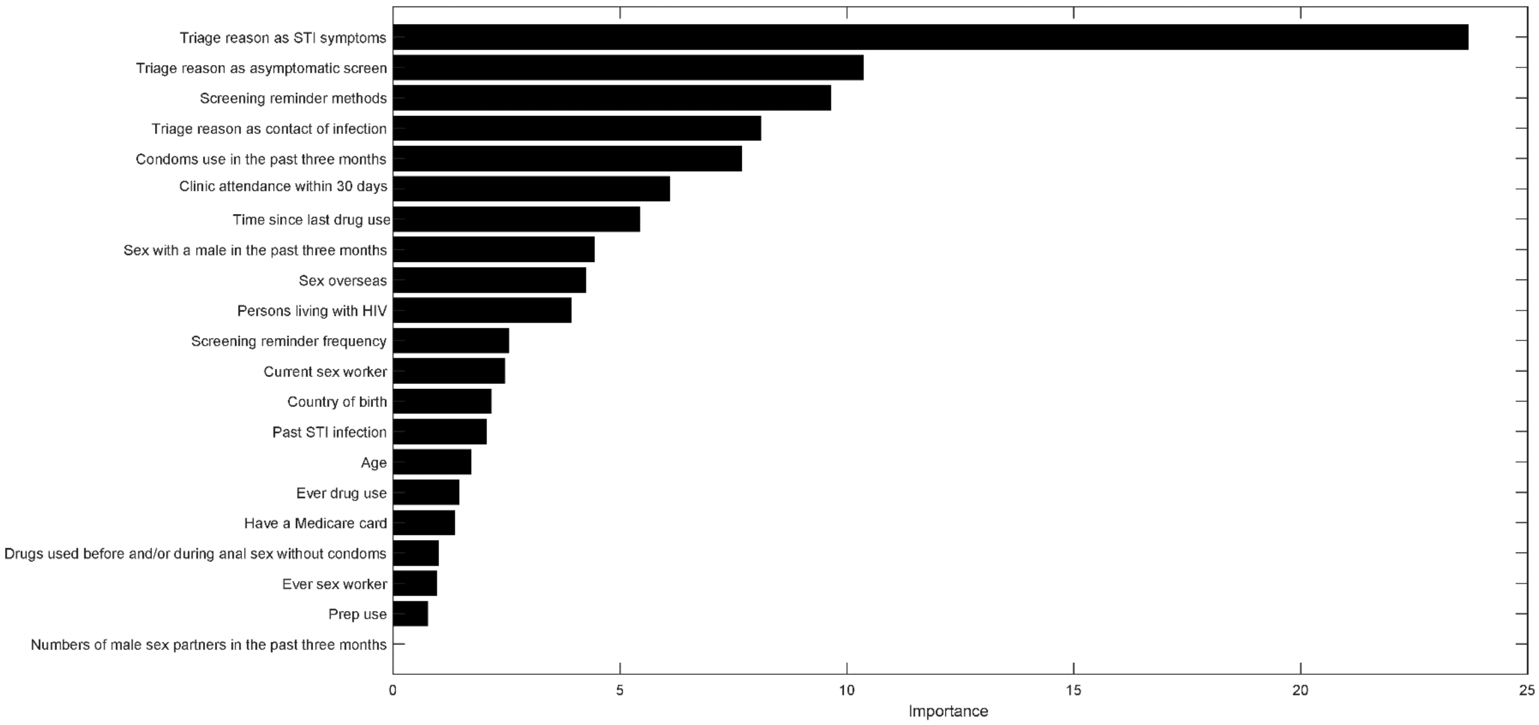

Supplementary Figure S14. Variable importance in the prediction of HIV/STI testing post clinic reminder message within one year by elastic net regression

Statistical analysis using multivariable stepwise logistic regression: An exploratory analysis

Multivariable stepwise logistic regression indicated that those whose reminder methods were only email reminders (adjusted odds ratio [aOR] 1.41, 95% confidence interval (CI) 1.16-1.72, vs. only SMS reminders), both email and SMS reminders (aOR 1.47, 95% 1.15-1.87, vs. only SMS reminders), PrEP use (aOR 1.29, 95% CI 1.06-1.55), and ever drug use (aOR 2.89, 95% CI 1.11-9.87) were more likely to revisit the clinic within 30 days after receiving a reminder message. Those who ever reported sex work (aOR 0.44, 95% CI 0.24-0.75) and Medicare cardholders (OR 0.83, 95% CI 0.69-0.99) were less likely to revisit the clinic within 30 days after receiving a reminder message**.**

Variables (Final stepwise logistic regression analysis of clinic attendance within 30 days)		N	Odds ratio	p
**Screening reminder methods**	SMS only	932	Reference	
	Email and SMS	603	1.46 (1.15, 1.87)	0.002
	Email only	1509	1.41 (1.16, 1.72)	<0.001
**Screening reminder frequency**	3 monthly	2528	Reference	
	12 monthly	35	0.80 (0.35, 1.68)	0.576
	6 monthly	481	0.79 (0.63, 1.00)	0.053
**Persons living with HIV**	HIV−	2572	Reference	
	HIV+	472	1.22 (0.96, 1.54)	0.102
**Triage reason as asymptomatic screening**	No	2314	Reference	
	Yes	730	1.20 (0.99, 1.46)	0.057
**Contact of infection**	No	2837	Reference	
	Yes	207	0.74 (0.52, 1.03)	0.083
**Past STI infection**	No	2029	Reference	
	Yes	1015	0.58 (0.49, 0.70)	<0.001
**Ever sex worker**	No	2947	Reference	
	Yes	97	0.44 (0.24, 0.75)	0.004
**Ever drug use**	No	32	Reference	
	Yes	3012	2.89 (1.11, 9.87)	0.050
**Prep use**	No	2256	Reference	
	Yes	788	1.29 (1.06, 1.55)	0.010
**Sex overseas in the past twelve months**	No	2175	Reference	
	Yes	869	0.87 (0.73, 1.04)	0.141
**Medicare Card holder**	No	881	Reference	
	Yes	2163	0.83 (0.69, 0.99)	0.043

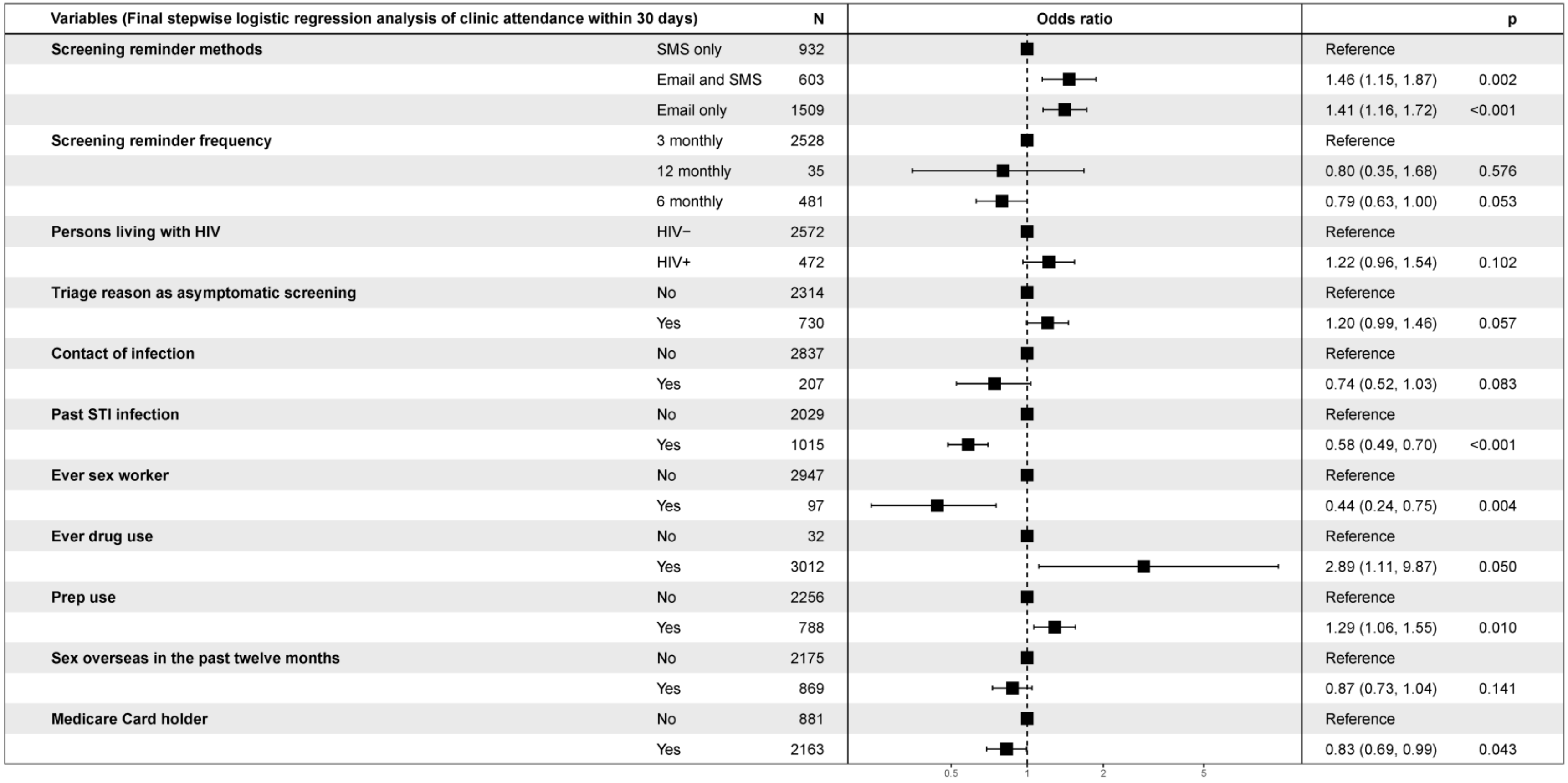

Supplementary Figure S15. Forest plot showing odds ratio (OR) and 95% Confidence Interval (CI) of stepwise multivariate logistic regression analysis in reporting timely clinic attendance

Multivariable stepwise logistic regression indicated that those whose clinic attendance reminder methods were the only email (aOR 1.97, 95% CI 1.19-3.27, vs only SMS reminders), both email and SMS reminders (aOR 1.89, 95% 1.03-3.53, vs only SMS reminders), triage reason as asymptomatic screening (aOR 59.18, 95% CI 12.82-1052.08)), triage reason as having STI symptoms (aOR 4.94, 95% CI 2.49-10.99), triage reason as having the contact of infection (aOR 13.69, 95% CI 2.78-248.90), and Medicare cardholders (aOR 1.70, 95% CI 1.06, 2.74) were more likely to have timely HIV/STI testing post clinic reminder message.

Variables (Final stepwise logistic regression analysis of HIV/STI testing within 30 days)		N	Odds ratio		p
**Type of screening reminder methods**	SMS only	223		Reference	
	Email and SMS	205		1.89 (1.03, 3.53)	0.043
	Email only	471		1.97 (1.19, 3.27)	0.008
**Persons living with HIV**	HIV−	747		Reference	
	HIV+	152		0.36 (0.23, 0.57)	<0.001
**Triage reason as asymptomatic screening**	No	660		Reference	
	Yes	239		59.18 (12.82, 1052.08)	<0.001
**Triage reason as symptomatic**	No	750		Reference	
	Yes	149		4.94 (2.49, 10.99)	<0.001
**Contact of infection**	No	850		Reference	
	Yes	49		13.69 (2.78, 248.90)	0.012
**Past STI infection**	No	674		Reference	
	Yes	225		1.71 (0.88, 3.51)	0.128
**Ever sex worker**	No	665		Reference	
	Yes	234		1.66 (0.98, 2.90)	0.067
**Medicare Card holder**	No	308		Reference	
	Yes	591		1.70 (1.06, 2.74)	0.029
**Numbers of male sex partners in the past three months**		899		1.07 (1.00, 1.19)	0.134

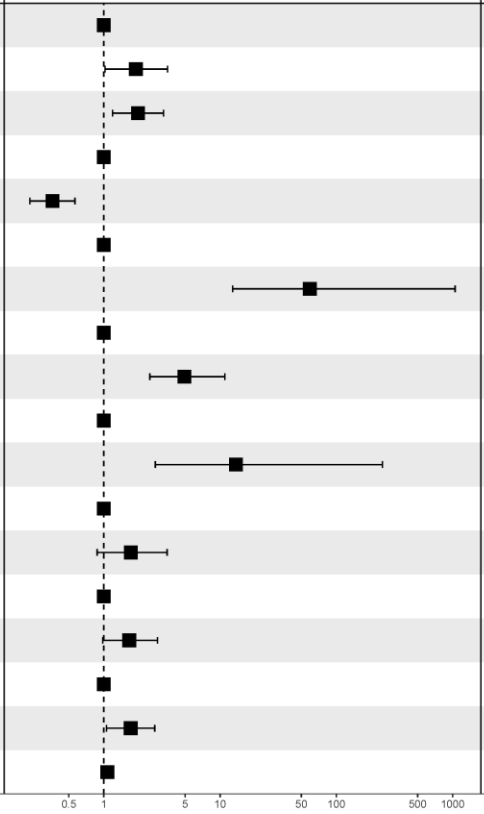

Supplementary Figure S16. Forest plot showing odds ratio (OR) and 95% Confidence Interval (CI) of stepwise multivariate logistic regression analysis in reporting timely HIV/STI testing after receiving a reminder message

Multivariable stepwise logistic regression indicated that those whose clinic attendance reminder methods were the only email (OR 3.12, 95% CI 2.33-4.19, vs only SMS reminders), both email and SMS reminders (OR 3.31, 95% 2.24-4.98, vs only SMS reminders), triage reason as asymptomatic screening (OR 25.89, 95% CI 10.84-84.64), having STI symptoms (OR 4.03, 95% CI 2.64-6.40), triage reason as having the contact of infection (OR 6.61, 95% CI 2.69-21.98), past STI diagnosis (OR 2.16, 95% CI 1.45-3.28), sex oversea in the past twelve months (OR 1.80, 95% CI 1.30-2.52), the numbers of male sex partners in the past three months (OR 1.08, 95% CI 1.03-1.16, per male sex partner increment), and older age (OR 1.01, 95% CI 1.00-1.03, per year increment) were more likely to the uptake of HIV/STI testing post clinic reminder message within one year. And six-monthly screening reminder frequency than three monthly reminder screening frequency (OR 0.64, 95% CI 0.47-0.89) and living with HIV (OR 0.46, 95% CI 0.34-0.623) were less likely to uptake HIV/STI testing post clinic reminder messages within one year.

Variables (Final stepwise logistic regression analysis of HIV/STI testing post clinic reminder message)		N	Odds ratio		p
**Clinic attendance within 30 days**	nope	2145		Reference	
	yes	899		0.47 (0.36, 0.61)	<0.001
**Clinic attendance reminder methods**	SMS only	932		Reference	
	Email and SMS	603		3.31 (2.24, 4.98)	<0.001
	Email only	1509		3.12 (2.33, 4.19)	<0.001
**Screening reminder frequency**	3 monthly	2528		Reference	
	12 monthly	35		0.98 (0.35, 3.53)	0.973
	6 monthly	481		0.64 (0.46, 0.89)	0.007
**Persons living with HIV**	HIV−	2572		Reference	
	HIV+	472		0.46 (0.34, 0.62)	<0.001
**Triage reason as asymptomatic screening**	No	2314		Reference	
	Yes	730		25.88 (10.84, 84.64)	<0.001
**Triage reason as symptomatic**	No	2462		Reference	
	Yes	582		4.03 (2.64, 6.40)	<0.001
**Contact of infection**	No	2837		Reference	
	Yes	207		6.61 (2.69, 21.98)	<0.001
**Past STI infection**	No	2029		Reference	
	Yes	1015		2.16 (1.45, 3.28)	<0.001
**Ever drug use**	No	32		Reference	
	Yes	3012		2.10 (0.85, 4.97)	0.095
**Prep use**	No	2256		Reference	
	Yes	788		0.73 (0.53, 1.01)	0.056
**Sex overseas**	No	2175		Reference	
	Yes	869		1.80 (1.30, 2.52)	<0.001
**Country of birth**	not Australia	1760		Reference	
	Australia	1284		1.26 (0.97, 1.66)	0.089
**Age**		3044		1.01 (1.00, 1.03)	0.035
**Numbers of male sex partners in the past three months**		3044		1.08 (1.03, 1.16)	0.007

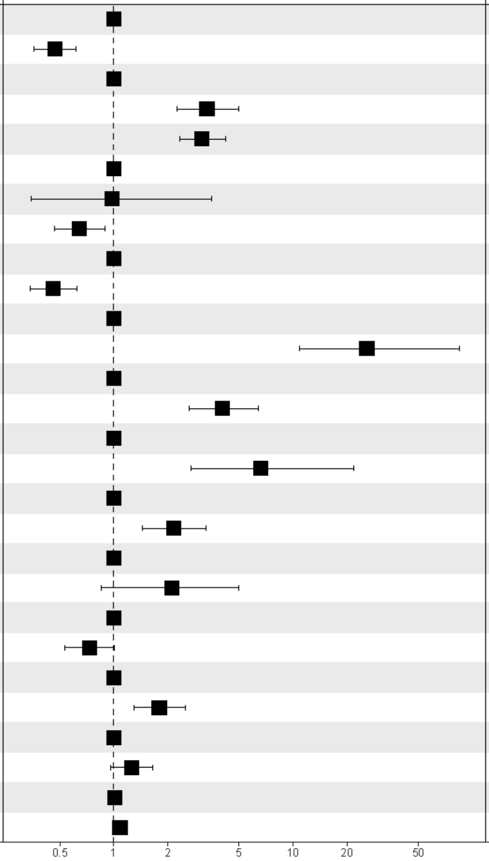

Supplementary Figure S17**.** Forest plot showing odds ratio (OR) and 95% Confidence Interval (CI) of stepwise multivariate logistic regression analysis in reporting HIV and STI testing post clinic reminder message within one year.
